# Influence of Inflammation in the Process of T Lymphocyte Differentiation: Proliferative, Metabolic, and Oxidative Changes

**DOI:** 10.3389/fimmu.2018.00339

**Published:** 2018-03-01

**Authors:** Marco A. Moro-García, Juan C. Mayo, Rosa M. Sainz, Rebeca Alonso-Arias

**Affiliations:** ^1^Department of Immunology, Hospital Universitario Central de Asturias (HUCA), Oviedo, Spain; ^2^Department of Morphology and Cell Biology, Institute of Oncology of Asturias (IUOPA), University of Oviedo, Oviedo, Spain; ^3^Facultad de Ciencias de la Salud, Universidad Autónoma de Chile, Talca, Chile

**Keywords:** inflammation, T lymphocytes, differentiation, metabolic reprogramming, exhaustion, redox balance

## Abstract

T lymphocytes, from their first encounter with their specific antigen as naïve cell until the last stages of their differentiation, in a replicative state of senescence, go through a series of phases. In several of these stages, T lymphocytes are subjected to exponential growth in successive encounters with the same antigen. This entire process occurs throughout the life of a human individual and, earlier, in patients with chronic infections/pathologies through inflammatory mediators, first acutely and later in a chronic form. This process plays a fundamental role in amplifying the activating signals on T lymphocytes and directing their clonal proliferation. The mechanisms that control cell growth are high levels of telomerase activity and maintenance of telomeric length that are far superior to other cell types, as well as metabolic adaptation and redox control. Large numbers of highly differentiated memory cells are accumulated in the immunological niches where they will contribute in a significant way to increase the levels of inflammatory mediators that will perpetuate the new state at the systemic level. These levels of inflammation greatly influence the process of T lymphocyte differentiation from naïve T lymphocyte, even before, until the arrival of exhaustion or cell death. The changes observed during lymphocyte differentiation are correlated with changes in cellular metabolism and these in turn are influenced by the inflammatory state of the environment where the cell is located. Reactive oxygen species (ROS) exert a dual action in the population of T lymphocytes. Exposure to high levels of ROS decreases the capacity of activation and T lymphocyte proliferation; however, intermediate levels of oxidation are necessary for the lymphocyte activation, differentiation, and effector functions. In conclusion, we can affirm that the inflammatory levels in the environment greatly influence the differentiation and activity of T lymphocyte populations. However, little is known about the mechanisms involved in these processes. The elucidation of these mechanisms would be of great help in the advance of improvements in pathologies with a large inflammatory base such as rheumatoid arthritis, intestinal inflammatory diseases, several infectious diseases and even, cancerous processes.

## Introduction

**Figure 1:**
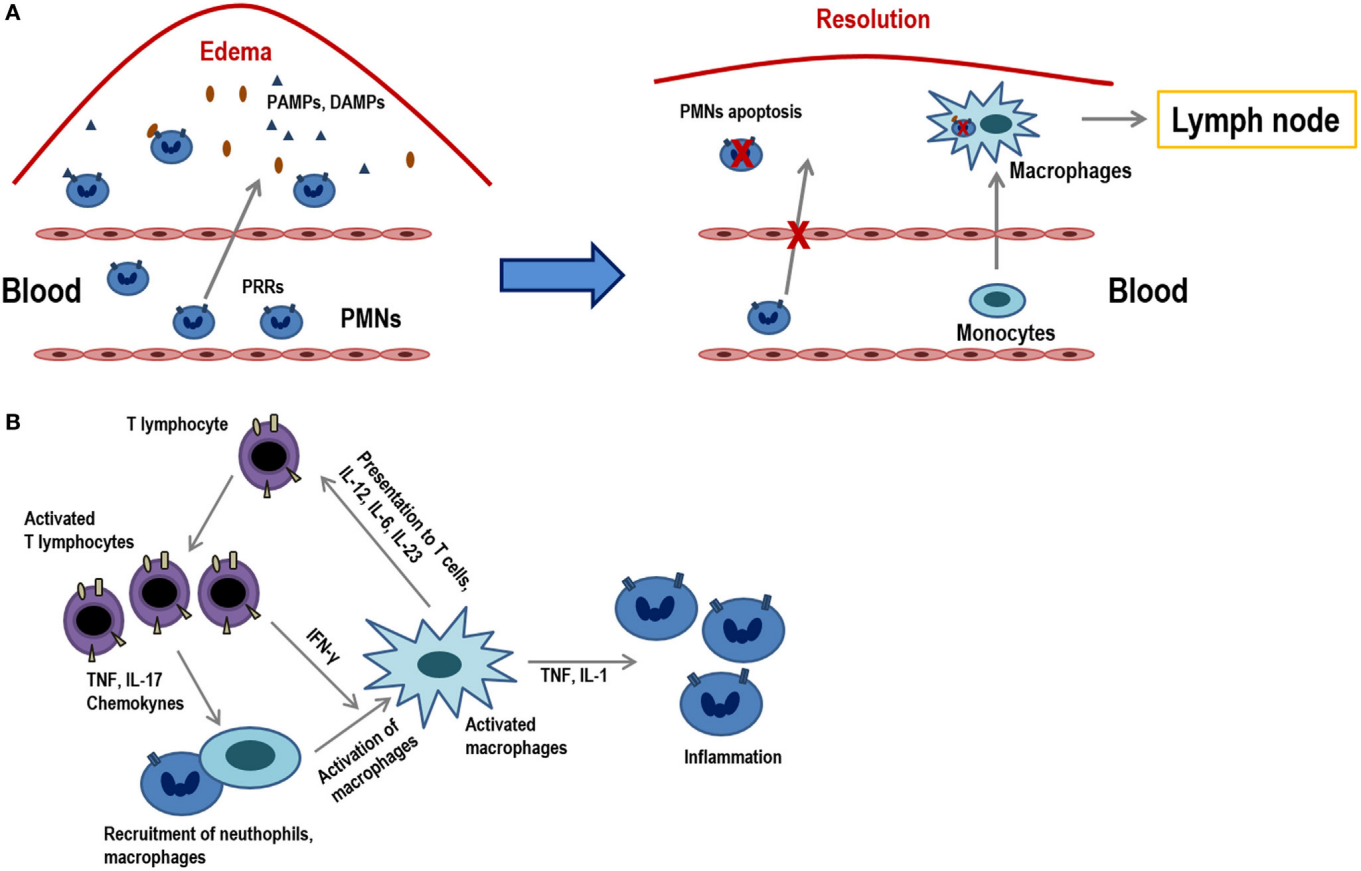
Acute inflammation vs. chronic inflammation. **(A)** The onset of acute inflammation is characterized by the accumulation of polymorphonuclear neutrophils (PMNs) and monocytes that rapidly convert into tissue macrophages, as well as the appearance of edema due to damage in the inflamed tissues. Damage-associated molecular patterns and pathogen-associated molecular patterns are recognized by pattern recognition receptors (PRRs) and attract PMNs to the place of infection. PMNs represent the first line of defense in infected tissues as they eliminate much of the pathogens or harmful materials by phagocytosis. When inflammation occurs, neutrophils suffer apoptosis and they are ingested by macrophages that migrate to the lymph nodes where they will present the antigens. **(B)** Activated T lymphocytes produce cytokines (TNF, IL-17, chemokines) that recruit macrophages and others (IFN-γ) which activate them. T lymphocyte subpopulations (Th1, Th2, Th17, etc.) produce diverse types of cytokines and, in turn, activated macrophages and stimulate T lymphocytes *via* the presentation of antigens and through different cytokines (IL-12, IL-6, IL-23). These macrophages also act on neutrophils by releasing molecules, such as TNF and IL-1.

Inflammation is the process in which leukocytes and plasma proteins are recruited from blood into tissues, accumulated and then activated to elicit an adequate immune response. Inflammation is triggered by recognition of pathogen-associated molecular patterns and damage-associated molecular patterns from injured tissues during innate immune responses and it is refined and prolonged during adaptive immune responses. Many of these reactions involve cytokines which are produced by dendritic cells, macrophages, and other types of cells during innate immune reactions. The leukocytes that are mainly recruited in inflammation are neutrophils, and monocytes (Figure 1A).

Inflammation can be sensed in the nearby lymph nodes and thus influence recruitment and activation of lymphocytes in the nodes. Peripheral tissue inflammation, which usually accompanies infections, causes a significant increase of blood flow into lymph nodes and consequently an increase in T lymphocyte influx into lymph nodes draining at the site of inflammation. T lymphocytes are fully activated only when a foreign peptide is recognized in the context of the innate immune system activation by a pathogen or by some other causes of inflammation. In this pro-inflammatory environment, co-stimulatory ligands and increase in the expression MHC class I and II molecules are induced in antigen-presenting cells (APCs), which are necessary for an optimal T lymphocyte activation to occur. There are also many inflammatory mediators and cytokines that attract T lymphocytes, activating them through their antigenic receptors (1).

Although innate immune stimuli may contribute to chronic inflammation, the adaptive immune system may also be involved because T lymphocyte-producing cytokines are powerful inducers of inflammation. In this scenario, macrophages are activated by type 1 helper T lymphocytes (Th1 cells), both through cell contact and through IFN-γ secretion (2).

When cells that responded to the inflammatory environment cannot eliminate pathogens, the acute inflammatory condition can become a chronic condition (Figure 1B). In addition to a local or systemic inflammatory status, this chronic phase is characterized by a maintained leukocyte infiltrate within the injured tissues. This low-grade inflammation is prevalent during aging and it is, therefore, denominated as “inflammaging.” Furthermore, this phenomenon can also be observed in chronic infections, autoimmunity diseases, other chronic inflammatory pathologies or cancer. Consequently, all of them are characterized by persistent antigens that induce a sustained inflammation concomitant with a marked differentiation of the adaptive immunity, mainly in T lymphocytes. These highly differentiated cells in turn contribute to perpetuate the process by producing increased levels of proinflamatory cytokines. During the last stages of differentiation, the pro-inflammatory environment may be responsible for an inefficient response of T lymphocytes, as it is shown in older individuals (3). Indeed, it has been demonstrated that a reduced cytokine-related JAK–STAT signaling is correlated with chronic inflammation and age-associated morbidities (4).

T lymphocytes are produced in the bone marrow from where they migrate to the thymus for completing the maturation process. Then, naïve T lymphocytes recirculate between blood and secondary lymphoid organs until they contact their specific antigen adequately and properly. Upon contact, they proliferate and acquire properties to assemble an appropriate immune response. After antigen elimination, part of these cells remains as memory cells, with its own homeostasis and proliferation, though most of the effective cells die. Memory cells display a series of migratory and functional features that allow them to mount a quick response after the reencounter with the antigen. Therefore, the adaptive immune response presents two main advantages for the individual. On the one hand, it allows to create a specific immune response against the invading pathogen, with which it will finish it in a very effective way. On the other hand, a set of memory cells is formed to endure for many years thus affording protection from new reinfection by the same pathogen.

T lymphocytes can be categorized using a combination of different surface markers (CD45RA, CCR7) in distinct groups depending on their functionality. These categories are the naïve (CD45RA+CCR7+), effector memory (EM, CD45RA−CCR7−), central memory (CM, CD45RA−CCR7+), and effector memory RA (EMRA, CD45RA+CCR7−) populations (5). The effector T lymphocytes are a quite heterogeneous population and the use of two markers (CD27 and CD28) allows categorizing this population into other subpopulations; (CD28−CD27−) is the population more differentiated of all (6).

The step from naïve T lymphocytes to effector and memory T lymphocytes is one of the most fundamental processes in the T lymphocyte-mediated immunity and requires proliferative, metabolic, and oxidative adaptations.

## T Lymphocyte Proliferation

### Naïve T Lymphocyte Homeostasis

**Figure 2:**
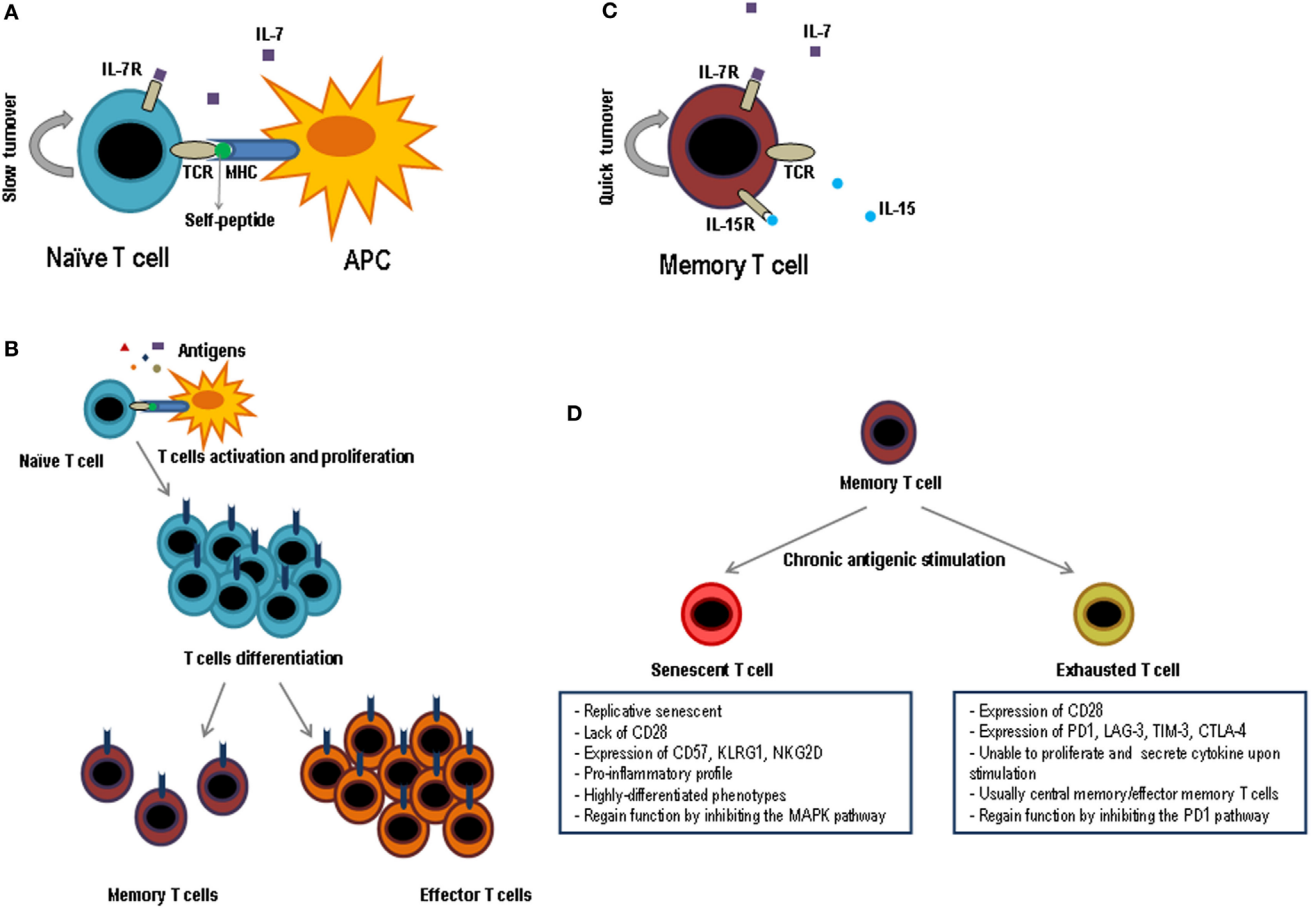
Functional phases of T lymphocytes. Schematic representation of the key factors for survival and maintenance of naïve cells **(A)** and memory **(C)**; while naïve cells are dependent on IL-7 and the contact with self-peptides by MHC, memory cells are dependent on IL-7 and IL-15 and do not need the contact with autoantigens. **(B)** After contact with its specific antigen, naïve T lymphocytes proliferate exponentially and give rise to two differentiated populations, effector T lymphocytes and memory T lymphocytes. Effector cells will be eliminated as soon as the pathogen is destroyed, while memory cells will last a long time and they will be responsible for protecting the individual against new infections caused by the same pathogen. **(D)** When the memory cells are subjected to multiple cycles of activation by means of chronic antigens they can generate two types of T lymphocytes, exhausted T lymphocytes and senescent T lymphocytes. The principal characteristics of both cell types are shown in the figure.

The number of naïve T lymphocytes remains stable in number and diversity along the time, when there are no involved powerful immune responses. However, T lymphocytes are neither a lethargic nor an immovable cellular population and indeed this naïve T lymphocyte pool is continuously interacting with other cells through homeostatic signs. The number of T lymphocytes in the periphery is almost constant although many new naïve cells appear every day from the thymus, especially during the early ages of the individual. Therefore, this homeostasis requires a strict control as it is very important to maintain this constant number along the time. Furthermore, the half-life of naïve T lymphocytes, roughly over 50 days, is quite longer than that of other cellular populations (7–9). Survival of naïve T lymphocytes requires signals mediated by the interaction of T lymphocyte receptor (TCR)–peptide–MHC and some cytokines, principally IL-7. This cytokine is particularly important for the correct T lymphocyte homeostasis but its concentration is very low. Thus, all the naïve T lymphocytes including the recent thymic emigrants (RTEs) compete for IL-7. If these cells do not receive enough IL-7 signal, they would die (Figure 2A). The necessity of soluble mediators to intervene in the homeostasis of naïve T lymphocytes has been evidenced by the capacity of various cytokines to avoid apoptosis of naïve T lymphocytes. Among these cytokines are IL-4, IL-6, lymphopoietin, and IL-7 (10–12), the latter being the one which plays a main role (13–15). Supporting this, when an IL-7 blocking antibody is injected or naïve cells are transfer into IL-7-deficient mice, survival of this subset is greatly diminished (13, 16–18). It is also believed that IL-7 is a limiting factor for determining the final size of the total lymphocyte pool, as it has been verified in several experiments in which the number of lymphocytes significantly increases in IL-7-overexpressing mice (19, 20). RTEs express low levels of IL-7 receptors, but they are more responsive to the cytokine than mature naïve T lymphocytes. Mechanistically, it has been demonstrated that IL-7 signaling in RTEs preferentially upregulates the antiapoptotic protein Bcl-2 expression, resulting in a decrease in cell apoptosis, but without an increase in proliferative effects. In contrast, mature naïve cells show a decrease in Bcl-2 expression. However, mature naïve cells have a greater proliferative response in the presence of IL-7 (21).

During lymphocyte development in the thymus, thymocytes reacting to self-molecules are eliminated or believed to be induced to produce regulatory T lymphocytes. But, on the other hand, in order to overcome positive selection, thymocytes must be able to recognize low affinity self-peptide–MHC so they can in turn leave the thymus as naïve mature cells (22). This reactivity to self-peptide–MHC is decreased after positive selection (23, 24), but does not disappear in naïve T lymphocytes, since the TCR ζ-chain continues to be phosphorylated and this can only occur if the TCR meets self-peptide–MHC complexes (25, 26). Therefore, consistent data from multiple studies confirm that self-peptide–MHC complex interactions are important for long-term naive T lymphocyte survival; however, these interactions raise much discussion and controversy. Even though there is a wide knowledge about the interaction between IL-7R and TCR in the homeostasis of naïve T lymphocytes, interestingly many of the routes that define the interactions between them are not well defined, but it is clear that it must include a mutual regulation between these two routes. This is a particularly interesting topic and should receive attention in further studies.

There are several works focused on the effect of acute inflammation, for example after bacterial sepsis, in the population of naive T lymphocytes. Most of these studies state that the function of these cells remains unchanged, but concomitant with a decrease in their count after being exposed to high concentrations of pro-inflammatory cytokines (27–29). A few recent studies have shown that the sensitivity of CD8+ T lymphocytes to antigens is greatly increased if cytokines, such as IL-12, IL-18, and IFN-γ, are present prior to antigenic recognition (30, 31). However, only a very few studies have approached the effect of persistent inflammation on naive T lymphocytes, as it occurs in the context of aging or in the pro-inflammatory tumor microenvironment. Most of them suggest that there is a loss in the number of naïve T lymphocytes and a decrease in their functionality, although it is not clear the molecular mechanisms involved and more studies must be carried out (3, 32).

### Clonal Expansion in Response to Specific Antigens

Naïve T lymphocytes present an amazing capacity to react against specific antigens through massive proliferation and differentiation to effector T lymphocytes. They are able to migrate to the infection sites and eliminate the triggering pathogen. Encounter with the antigen takes place in the secondary lymphoid organs, where APCs show them to the T lymphocytes. Then, a differentiation process begins; it is destined to produce large amounts of effective cells to fight against the pathogen with a clonal proliferative process but without coming to exhaustion. Interaction between T lymphocytes and APCs continues in tissues and so does the expansion and cellular differentiation, in an effort to contain the infection without damaging the tissues of the affected individual. Therefore, antigenic recognition by T lymphocytes provides a series of changes that leads to a clonal expansion of differentiated and effective T lymphocytes. The activation of T lymphocytes through its specific TCR can be detected seconds after antigenic contact, and it continues during hours and days in the context of pathology (33, 34) (Figure 2B).

The activation of lymphocytes after recognizing a peptide in the context of a MHC molecule (priming of naïve T lymphocytes) carries a series of processes of various types (genetics, proliferative, differentiation, biochemical) that lead to the formation of specific clones of effective lymphocytes, some of which will remain for long time in the form of memory cells. Before antigen exposure, the frequency of naive T lymphocytes specific for any antigen is 1 in 10^5^ to 10^6^ lymphocytes. After antigen exposure, the frequency of CD8+ T lymphocytes specific may increase to as many as 1 in 3 CD8+ T lymphocytes, representing a >50,000-fold expansion of antigen-specific CD8+ T lymphocytes, and the number of specific CD4+ cells increases up to 1 in 100 CD4+ lymphocytes may increase up to 5,000-fold.

It is well known that the single contact of T lymphocyte with its antigen is not enough to generate a cellular response but it rather causes the cell to enter into a refractory state and does not respond to any stimulus (35). This discovery led to the hypothesis that there should be other additional stimulatory signals that would enable T lymphocytes to become activated and exert their functions. Thus, when the CD28 molecule was identified as a co-stimulator of T lymphocyte function, the theory of the “two signals” was then reported, these two signals being necessary for the T lymphocytes activation. However, numerous evidences have suggested that other membrane-bound and soluble inflammatory signals are necessary to complete the activation of T lymphocytes, thus enforcing the “three signals” and “four signals” alternative theories. All experimental data seem to highlight the significant role of inflammatory mediators in the process of T lymphocytes differentiation into the effector population, resulting in a more adequate tool to respond to the aggression suffered (36, 37). On the other hand, there is also a great deal of data suggesting that TNF superfamily receptors (CD30, 41BB, OX-40, CD27) interact with their ligands in APCs (CD30L, 41BBL, OX-40L, CD70), which favors the survival of the activated cells and their passage into memory cells (38, 39).

T lymphocytes also have a large number of inhibitory molecules that help to regulate the cellular response, in a way that keeps this response not to be exaggerated and that would end up being harmful to the body. These inhibitory molecules act both, by limiting the co-stimulatory signals and by binding to the appropriate co-stimulatory receptors. The main inhibitory receptors belong to the CD28 family, including cytotoxic T lymphocyte antigen 4 (CTLA-4) and programmed death 1 (PD-1), both being involved in the phenomenon of tolerance. CTLA-4 is an inhibitory molecule expressed in activated T lymphocytes which causes an increase in the intracellular phosphatase activity, thus producing a decrease in the signals generated by the TCR and CD28 molecule. On the other hand, CTLA-4 acts also as a competitive receptor for the CD80/CD86 receptor, but with a higher affinity for these receptors than CD28 itself (40, 41). As a result, depending on their level of expression on the cell surface, CTLA-4 may interfere with CD80/CD86 binding. Several more inhibitors, such as lymphocyte activation gene 3, and V-domain Ig suppressor of T lymphocyte activation have been described, blocking these inhibitors by specific antibodies are being studied to increase the immune response in numerous cancers (42, 43).

**Figure 3:**
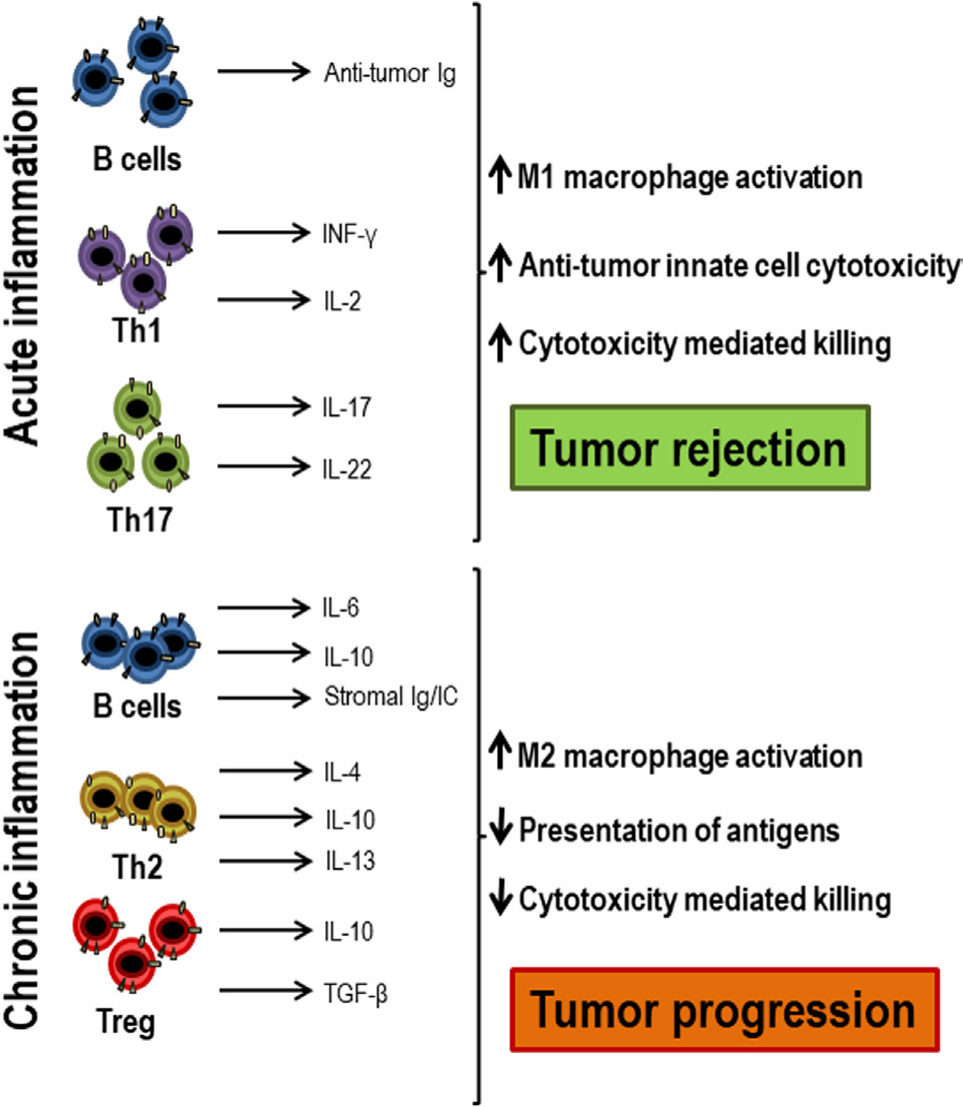
Acute and chronic inflammation participation in antitumor responses. When acute inflammation is activated by tumor cells, Th1 cells secrete antitumor cytokines, such as IFN-γ and IL-2, which, together with antitumor antibodies produced by B lymphocytes, exert an antitumor response and lead to tumor rejection, attracting innate immune cells and cytotoxic T lymphocytes. However, when chronic inflammation occurs in response to tumors, there is often an increase in regulatory T lymphocytes, Th2 cells, and activated B lymphocytes that secrete growth factors, such as IL-4, IL-6, IL-10, IL-13, TGF-β, and immunoglobulins that decrease both antigenic presentation and the activation of cytotoxic cells, favoring tumor progression.

The scenario in which the T lymphocyte response occurs may change in situations such as aging or in tumor microenvironment, where systemic or local inflammation is present (Figure 3). The levels of pro-inflammatory cytokines, such as IL-6, IL-1β, TNF-α, or GM-CSF, increase in these cases and this influences the lymphocyte response (3, 44). Dendritic cells are essential for T lymphocytes to be activated and differentiated as they present the antigen, producing co-stimulatory signals and essential cytokines. All these processes are highly dependent on the inflammatory environment mainly in chronic situation (45, 46). In the immune response during aging, myeloid dendritic cells in inflammatory environments have a decreased ability to present the antigens to CD4+ and CD8+ T lymphocytes (47). Moreover, most studies describe a reduced ability to produce cytokines by dendritic cells stimulated through toll-like receptors *in vitro*, a defect related to low response to vaccination in elderly (48, 49). TNF-α directly affects the immune response, in part by reducing the expression of the co-stimulatory molecule CD28 (50, 51). The pro-inflammatory environment may also be responsible for the attenuated response of T lymphocytes to cytokines, possibly due to the activation of negative regulatory pathways (4). According to the inflammatory environment in which lymphocytes are, the polarization of T lymphocytes will be different. In inflammatory environments, there is no evidence that Th1, Th2, or Th17 cells are diminished, but follicular T lymphocytes have been found to be less common in the elderly with systemic inflammation in response to vaccination (52, 53). It has been shown that CD4+ T lymphocytes activated in inflammatory environments are less responsive to type I interferons due to recruitment by the IFNR signaling complex of SHP1 (54). In conclusion, the inflammatory environment must be taken into account when evaluating immune responses. Individuals with high systemic and/or local inflammatory levels, as it occurs in the elderly and in cancer patients, may have an impaired response to cytokines and a poor response to antigens. In these cases, an anti-inflammatory treatment could be beneficial in trying to restore a correct immune response.

### Maintenance of Memory T Lymphocytes

The T lymphocyte-specific antigen response is characterized by clonal expansion, followed by a contraction in the number of specific cells and the formation of memory T lymphocytes. In this process, T lymphocytes acquire many key features to afford protection against re-infections, which are potentially deadly. Memory T lymphocytes are found in greater numbers than naïve T lymphocytes and in the presence of IL-7 and IL-15 these cells can be maintained for long periods of time without antigenic stimulation (55, 56) (Figure 2C). Central and effector memory T lymphocytes are less dependent on the contact with the MHC–peptide complex for survival than naïve T lymphocytes do (57). In addition, their self-renewal is three to four times faster than in naïve T lymphocytes and has a high proliferation rate in lymphopenia (58). The large number of memory cells, their high ability to reactivate, produce cytokines, and kill after antigenic stimulation and their distribution by almost all tissues makes memory T lymphocytes capable of protecting the individual much better than naïve T lymphocytes.

As previously mentioned, memory T lymphocytes can be divided into two populations, effector memory cells (EM, CD45RA−, CCR7−) and central memory cells (CM, CD45RA+, CCR7+), but other subpopulations can be established from the expression of CD27 and CD28 (5). EM are preferentially located in non-lymphoid and mucosal tissues and have a lower response threshold than CM, which are found mainly in secondary lymphoid organs and have a greater expansion capacity than EM.

There is no unanimity to describe the way by which T lymphocytes become effector and central memory cells (59). The most convincing hypothesis suggest a developmental trend from naïve T lymphocytes to memory cells, in which most memory cells have gone through a phase of effector cells. Some effector cells might then evolve into memory cells since some microarray studies have shown that passage of naïve cells to effectors and memory is a gradual step (59–61). Besides, several laboratories have used murine models of labeled effector T lymphocytes to track them, showing that most memory cells have indeed derived from these effector cells (62, 63). Thus, at least a set of memory cells that appear after an infection are generated from the effector cells.

Numerous studies have shown that exposure to a restricted inflammation increases the appearance of memory-like T lymphocytes when the intensity of inflammatory signals is controlled (59, 64). Several inflammatory molecules, such as interferon type I and IL-12 can be considered to act as the third signal (in addition to TCR and co-stimulation) and thus, to promote differentiation of T lymphocytes to effector cells (65).

According to several studies, prolonged and/or intense exposure to the pro-inflammatory cytokine IL-12 promotes a preferential differentiation toward effector cells, rather than memory cells (66, 67). The effect of other inflammatory molecules on T lymphocytes may be indirect. For instance, an interesting study found a blockage in the contraction of T lymphocytes when there was a deficiency of IFN-γ (68). In this scenario, we believe that IFN-γ offers a competitive advantage for the appearance of memory T lymphocytes, in some types of immune responses (69). Some studies suggest that at least some level of inflammatory signal is necessary for the differentiation to memory T lymphocytes. T lymphocytes deprived of this inflammatory signal (third signal) or deficient in T-bet transcription factor showed a decreased ability to produce long-lived memory cells. T-bet is needed for the expression of the CD122 molecule, that is the beta chain of the cytokine receptors IL-15 and IL-2, and to be able to react to homeostasis mediated by IL-15 (66, 70, 71). The cytokine IL-15 not only can promote the division and proliferation of differentiated and memory T lymphocytes but it is also capable of increasing its functional capacities (72, 73).

The differentiation of memory T lymphocytes in the course of chronic infections or in the presence of persistent antigens (autoimmune diseases, tumors, or atherosclerotic diseases) is different from differentiation in acute infections and leads to a defect in the functionality of T lymphocytes. These cells phenotypically acquire expression of several typical cytotoxic cell markers, such as NKG2D, PD-1, CD56, CD16, KLRG1, etc. (74, 75). Unlike memory cells generated after the elimination of an acute infection, memory cells in situations where the antigen is present in a chronic form, and in an inflammatory environment, proliferate and increase in number because the continuous stimulus (76, 77). These subpopulations, in turn, have propensity to secrete pro-inflammatory cytokines, such as IFN-γ, IL-1, TNF-α, and IL-6, contributing to increase the systemic or local inflammation (78).

### New Encounters with Specific Antigens and Telomere Maintenance

One of the most remarkable characteristics of the immunological memory system is the capacity of the antigen-primed T lymphocytes to carry out a rapid response after they encounter the same antigen again (79). Co-stimulation signals have been recognized as critical for optimal T lymphocyte responses and result from important interaction between receptors on the surface of T lymphocytes and their ligands on APCs. However, memory T lymphocytes exhibit more reduced dependency to co-stimulation, and a significant lower threshold to respond.

T lymphocytes must be able to grow exponentially upon the first, and especially the second and the following, encounter with an antigen, and when they are no longer needed some of them enter apoptosis and disappear. Like most normal cells, lymphocytes are able to pass through a limited number of division cycles. Limited number of cell cycle divisions is associated to cell senescence or cell stress, which ends up in cell death triggering. Similar to what occurs to normal cells that evolve toward a neoplastic state acquiring a high rate of proliferation, lymphoid cells are also able to constitutively activate telomerase (80). Upregulation of telomerase and consequently elongation of telomeres allows extending the cells lifespan (81, 82). When the mechanisms that compensate telomeric shortening disappear and when shortening reaches a critical point known, cells enter a state of growth arrest termed senescence (83, 84). Telomere size can be determined by analyzing the terminal restriction fragments (TRF) that contains the TTAGGG region. The length of the TRF varies depending on cell population and each chromosome within the same cell. The critical point for cells to enter senescence appears when the size of TRFs reach less than 6 kb (85). The overall finding from different studies is that T lymphocytes in humans can carry out a certain number of divisions, after which they can no longer be divided (86, 87). Importantly, the reach of the replicative senescence stage by T lymphocytes does not imply a loss of cell viability. Moreover, senescent T lymphocytes under suitable conditions can remain alive and metabolically active for a prolonged period of time (88, 89). This transitional state of lymphocytes is not, however, shared with stem cells or malignant tumor cells, which do not reach replicative senescence and have stable chromosomes despite intense division. Stem and malignant T lymphocytes maintain telomerase activity and, therefore, replicative capacity throughout their lives (90). Nevertheless, by using mice lacking telomerase, it has been demonstrated that telomere shortening shunts premalignant T lymphocytes into the senescent state, therefore, reducing tumorigenesis (91).

It is very important to distinguish undifferentiated and highly differentiated T lymphocytes in order to study telomerase activity in these two populations. The undifferentiated T lymphocytes (CD27 +CD28+) display longer telomeres than highly differentiated T lymphocytes (CD27−CD28−) while intermediate populations (CD27−CD28+ or CD27+CD28−) have telomere lengths between both, undifferentiated and highly differentiated cells (92, 93). In addition, the proliferation ratio in T lymphocytes is higher in highly differentiated CD45RA−CCR7− cells that have a lower telomeric length (92, 94). Telomerase activity correlates with the length of telomeres, thus this activity is greater in the more undifferentiated cells and much lower in the cells with high differentiation. In addition, as the cell ages, the ability to induce telomerase expression and activation is lost (92, 93, 95). Moreover, differences in the behavior of telomerase have been found between CD4+ and CD8+ T lymphocytes. Cultures of CD4+ and CD8+ T lymphocytes from the same subject that have encountered an antigen for the fourth time were unable to increase telomerase production. However, the CD4+ T lymphocytes had much higher telomerase activity than that of the CD8+ T lymphocytes from the same donor (96). Several studies have shown that homeostasis of CD4+ T lymphocytes is much more rigorous than that presented by CD8+ T lymphocytes. In addition, aging in lymphocytes was previously described in CD8+ T lymphocytes, since the changes observed in immunosenescence occur much earlier in these CD8+ T lymphocytes (97–99).

The signaling *via* the TCR and the co-stimulation with other molecules such as CD28 is essential for the induction of telomerase activity. This activity peaks 4–5 days after the TCR has been stimulated, but presents a decrease in its activity after 10 days (92, 100, 101). T lymphocytes can proliferate under stimulation of various cytokines without the mediation of TCR. This is homeostatic proliferation, the mechanism by which naïve and memory T lymphocytes are maintained in the periphery (8). The cytokines IL-7 and IL-15 have been related to telomerase activity in the CD4+ and CD8+ T lymphocytes, respectively (102, 103). Our laboratory has demonstrated that IL-15 has a preferential effect on the CD4+CD28− T lymphocyte population, which causes an increase in proliferation and specific responses of these cells (73). It has also been found inhibitory effects on telomerase activity by some cytokines such as IFN-α and TGF-β (104, 105).

Factors that promote repeated T lymphocyte stimulation, such as persistent antigen and chronic inflammation, appear to drive telomere loss and replicative senescence. Elderly individuals and cancer patients, often exhibit chronic inflammation characterized by immune system dysregulation with increased inflammatory cytokine production (73, 74, 106, 107) and redox imbalance due to reduced antioxidant defenses and overproduction of reactive oxygen species (ROS) (108). It is now recognized that chronic inflammation is a major risk factor for several age-associated diseases, including chronic obstructive pulmonary disease, neurodegeneration, obesity, and vascular disease (109, 110). Premature telomere erosion in peripheral blood lymphocytes is a common characteristic of these diseases as well as autoimmune syndromes (103). These findings suggest that telomere loss increases susceptibility to autoimmune disease and may be a predisposing factor for age-related inflammatory disease.

Restoring telomerase activity in T lymphocytes would have a great impact on human lives by restoring telomere shortening and, in turn, avoiding the deleterious effects of aged T lymphocytes. Several studies have shown that if the telomerase activity is preserved, the telomeric length is stabilized and replicative senescence can be delayed (111, 112). One possible solution to stimulate telomerase activity would be to eliminate senescent T lymphocytes from the bloodstream or bring them into apoptosis. Telomere loss could be stopped by inhibiting cytokines, such as TNF-α, mediating telomere shortening.

### T Lymphocyte Exhaustion

In a viral infection or cancerous environment where there is a permanent exposure to certain antigens and high inflammation, memory cells are highly affected (113). This alteration, known as T cell exhaustion, presents a series of characteristics: progressive loss of effector functions, upregulation and co-expression of many inhibitory receptors, alteration of transcription factors, dysfunctional metabolism, failure to enter a quiescent state, and response to normal homeostasis (114) (Figure 2D). Although the appearance of exhausted T lymphocytes was first described in an environment of viral infection, it has also been observed in the presence of cancer and in various inflammatory diseases. Initially, exhausted T lymphocytes have a defect in proliferative progression and a defect in the expression of telomerase, but the rest of their functions are completely conserved (115). Next, exhausted T lymphocytes, mainly due to continuous antigenic stimulation and a pro-inflammatory environment, enter into a state of differentiation where they gradually lose their effector functions, such as cytokine production and cytotoxic capacity, which prevents these cells from being effective against cancer or microbial infections (113). Exhausted T lymphocytes normally arise during high-grade chronic infections in highly pro-inflammatory environments, where the level and duration of antigenic stimulation are critical for this process to occur (116, 117).

Viral infections and cancerous tumors can cause the appearance of exhausted T lymphocytes, but not all cases lead to an exhausted T lymphocyte (113, 118, 119). The difference in the ability to produce exhausted T lymphocytes may be due to different T lymphocyte specificities. The most effective specificities in stopping pathogens inactivate them faster and destroy them quickly. Exhausted cells regain functionality and become typical memory T lymphocytes when viral infection is under control and in turn, antigen concentration decreases (120).

Exhaustion of T lymphocytes is accompanied by an increase in expression of inhibitory molecules, including PD-1, CTLA-4, LAG3, 2B4, CD160, and T lymphocyte immunoreceptor with immunoglobulin and ITIM domains (TIGIT). PD-1 is an inhibitory protein that intervenes in self-tolerance by inhibiting the activation of T lymphocytes using a similar mechanism as describe for CTLA-4 (121). After PD-1 interaction with its ligands, namely PD-L1 or PD-L2, TCR signaling is blocked by recruiting SHP-2 phosphatase and subsequent dephosphorylating of the antigen receptor (122). Interestingly, both ligands are often overexpressed in many tumor cells, but PD-1 is highly expressed in T lymphocytes from patients with different types of cancer. PD-1 ligand levels from tumor cells and PD-1 levels from T lymphocytes are usually correlated with the tumor aggressiveness and poor prognosis (121).

The proliferation of exhausted cells is partially compensated with PD-1 suppression, but telomerase activity is not restored (123). Many causes leading to T lymphocyte exhaustion are shared with those leading to T lymphocyte aging. Several epigenetic studies in naïve and central memory T lymphocytes reveal an age-associated loss of access of T lymphocytes promoters to their chromatin site of action, particularly to the NRF1-binding sites (124). This loss seems to intervene in the reduced expression of the genes of the mitochondrial respiratory chain and, therefore, in the deficient oxidative phosphorylation that can lead to death of the T lymphocytes (125). Repression of the molecule PGC1α, a cofactor of NRF1 activity, is an event that appears early in exhausted T lymphocytes, indicating a mechanistic overlap between T lymphocyte aging and exhaustion (126). Effector memory T lymphocytes of elderly individuals compared to effector memory cells in young people show few differences with respect to the accessibility of chromatin promoters. Thus, the memory T lymphocytes in elderly individuals do not exhibit the epigenetic marks of the exhausted T lymphocytes (124, 127). This observation may indicate that T lymphocyte aging affects mainly to naïve cells and central memory, possibly through pathways similar to those involved in T lymphocyte exhaustion. On the other hand, effector T lymphocytes in the elderly do not show any signs of exhaustion.

The epigenetic phenotype of the exhausted T lymphocytes remains stable when PD-1 is blocked and the restoration of function is, therefore, only transient in most T lymphocytes (128). This evidence leads us to consider whether treatment with PD-1 blockers decreases along with age due to aging of the exhausted T lymphocytes or rather because the responsive fraction of exhausted T lymphocytes to treatment decrease with age.

The appearance of exhausted T lymphocytes prevents the possibility of an adequate control of infections and malignant tumors. Therefore, if overexpressing pathways in the exhausted T lymphocytes could be modulated, for example, by inhibiting PD-1 and CTLA-4, their dysfunctional state could be reversed and immune responses invigorated.

## Metabolic Reprogramming

T lymphocytes must carry out metabolic strategies throughout their differentiation process and because of the changing microenvironment in which they are located. The purpose of these adaptations is to meet energy and structural needs in the different stages of proliferation and to achieve functional responses according to the availability of nutrients.

Naïve T lymphocytes, since they exit the thymus as mature cells and during their travel throughout the secondary lymphoid tissues until encountering their specific antigen, show a reduced rate of cell division. Even more, it is considered that they are in a functional quiescence, which does not require a high-energy consumption. They use the available nutrients trying to obtain the highest energy yield. Glucose, fatty acids, and amino acids are metabolized until they enter into the tricarboxylic acid (TCA) cycle where ATP and reducing equivalents are generated, which subsequently increase the production of ATP upon entering the pathway of oxidative phosphorylation (129, 130).

When naïve T lymphocytes encounter APCs carrying their specific antigenic peptide, activation takes place in a lymph node. Clonal expansion of the antigen-specific T lymphocytes is mainly mediated by IL-2 and the subsequent functional differentiation adapts the response to the specific triggering pathogen. Proliferation leads to generate sufficient amount of specific T lymphocytes capable of eradicating the pathogen. Thus, T lymphocyte activation not only requires energy but also the production of precursors that support the explosive proliferation through the biosynthesis of the required cellular components, proteins, lipids, nucleic acids, etc. These processes imply a high increase in energy demands, resulting in a switch in the metabolic pathways of nutrient utilization. Initially, there is an increase in glucose uptake, by increasing the expression of glucose transporter (GLUT) such as GLUT-1 (131). Glucose is, therefore, essentially metabolized to lactate by aerobic glycolysis, a phenomenon known as “Wargurg Effect,” which was initially described in tumor cells and, more recently, also in T lymphocytes (132–134). It is currently considered that “T lymphocyte activation-induced metabolic reprogramming is reminiscent of the metabolic changes associated with oncogenic transformation” (135, 136). More than 90 years ago, Otto Warburg described that tumor cells, even in the presence of oxygen, showed a high rate of glycolysis. This so-called aerobic glycolysis is now recognized as one of the new hallmark of cancer capabilities (5). In differentiated cells, glucose renders through the TCA cycle an energetic yield of 36 molecules of ATP. And yet, cancer cells rather prefer to compromise a high ATP production in order to obtain other benefits, in terms of building blocks for their new daughter cells (137–139). Recently, a similar program has also been observed in all proliferative cells including T lymphocytes upon antigen activation. This metabolic adaptation ensures a high glycolytic rate, which is not inhibited by the production of mitochondrial ATP. Moreover, glycolysis inhibits apoptosis and contributes to the maintaining of mitochondrial membrane potential (137, 140). Regarding the immune system, metabolic reprogramming has also been linked to the acquisition of certain functional properties by T lymphocytes such as the secretion of IFN-γ (134, 141).

Other functional parallelism found is glutaminolysis, which also increases in response to T lymphocyte activation as well as after transformation of cancer cells. Glutamine is rapidly consumed by tumor cells, playing a key structural role as a nutrient in the biosynthesis of nucleotides. The concentration of glutamine has been shown to be limiting in the progression of the cell cycle. Glutamine deprivation leads to cell cycle arrest in some cell types. Glutamine is not only an important source of carbon and nitrogen for the synthesis of other amino acids but the α-ketoglutarate generated is an anaplerotic substrate of the TCA cycle. Thus, it may contribute to the generation of ATP when glucose is primarily derived toward this so-called aerobic glycolysis (142).

On the other hand, pentose phosphate pathway (PPP) is the main catabolic route that generates ribose, necessary for the synthesis of nucleotides, and NADPH, essential for proliferation as it provides the reduction equivalents for fatty acid and cholesterol biosynthesis. Furthermore, PPP plays an essential role in balancing the redox status by regulating the production of glutathione (143). The utilization of PPP is usually elevated in cancer cells and in proliferative T lymphocytes. The key enzymes in the pathway are overexpressed in cancer and oncogenes and tumor suppressors have been shown to regulate PPP activity.

Metabolic decisions are dependent on the co-stimulatory signals received by T lymphocytes, in particular on CD28, which mediates many of these processes *via* the PI3K–Akt–mTOR pathway. However, in cancer cells intracellular programs combine with extracellular signals, to achieve a significant independence from external requirements. Genetic alterations as common as PI3K and its negative regulator PTEN as well as gen amplifications of upstream receptor tyrosine kinases result in an increase in glucose uptake and in the metabolic reprogramming in several cancer cells (144). It is noteworthy to mention that the metabolic state of cancer cells has a potential influence on the surrounding cells. Thus, within the tumor microenvironment, cancer cells alter the metabolic composition of the extracellular milieu, affecting the signaling pathways that influence the infiltration of immune cells, including T infiltrating lymphocytes. mTOR is a central part of various signals that come from the immune microenvironment because mTOR works as a sensor of extracellular medium conditions. mTOR is an evolutionarily conserved serine/threonine kinase, which forms two complexes, mTORC1 and mTORC2, determined by the association with different adapters and scaffolding proteins. mTOR is responsible for integrating different responses upon receiving environmental signals and for the control of various cellular functions, such as growth, apoptosis, actin reorganization, metabolism, and ribosome genesis (145, 146). mTOR activation targets T lymphocyte metabolism, switching it toward glycolytic metabolism by induction of two major transcription factors, namely HIF1α and c-MYC (147, 148). HIF1α is stabilized under hypoxia but it can also be activated by mTOR even under aerobic conditions. It increases the expression of GLUTs and glycolytic enzymes, such as pyruvate dehydrogenase kinase 1, limiting the entry of pyruvate into TCA cycle and favoring its reduction to lactate. The deregulation of multiple elements of the mTOR pathway has been reported in many types of cancers, implying significant effects on tumor progression. Likewise, mTORC1 signaling controls transcription of many genes, some of which are involved in metabolic and biosynthetic pathways (149).

Signals derived from anaerobic conditions and nutrients availability may modulate the cytokine profile of T lymphocytes (150). CD4+ T lymphocyte differentiation into a specific effector phenotype (mainly Th1, Th2, Th17, Treg) will depend fundamentally on the cytokines present in the immunological microenvironment at the time of antigenic presentation. mTOR plays a key role in the differentiation into the effector phenotypes, but not into Treg, so mTOR1 is needed for differentiation toward Th1 and Th17, whereas mTOR2 regulates Th2 differentiation (151). Thus, mTOR coordinates the metabolic pathways and the differentiation of each subpopulation of CD4+ T lymphocytes (146). High levels of HIF1α direct the CD4+ T lymphocyte metabolism to the glycolytic pathway, favoring the activation of RORγt transcription factor and its differentiation toward a Th17 inflammatory phenotype. Inhibition of this pathway, even under conditions that promote Th17 differentiation, results in Treg lymphocytes (152, 153). Th1 also possess a high glycolytic rate, concomitant with a higher surface location of GLUT1 (154). In fact, there is a coordinated regulation between T lymphocyte metabolism and the IFN-γ production by Th1. GAPDH is attached to UA-rich regions located at the 3′ in the untranslated region of the IFN-γ mRNA in non-activated cells. When glycolytic metabolism is activated, this enzyme is involved and does not bind to the mRNA, allowing its translation and IFN-γ production (134).

It has also been shown that the limited availability of glutamine in the extracellular medium favors Treg differentiation, even in the presence of cytokines involved in Th1 differentiation (155). The reason seems to be the decrease in intracellular levels of α-ketoglutarate, a mTORC1 activator, the expression of T-bet transcription factor and, as a result, differentiation into Th1. Similarly, the production of GLUT1 receptor, essential for the development of Th1 responses, is increased, whereas Tregs are unaffected by deficiency in this receptor and maintain their inhibitory capacity on T lymphocytes independently of GLUT1 (156).

In CD8+ T lymphocytes, mTORC1–HIF1α is activated by a PI3K–Akt-independent pathway, through phosphoinositide-dependent kinase 1. Metabolically, it promotes the activity of glycolytic enzymes and at functional level, it activates cytolytic capacity, controls the migration, and inhibits the generation of memory cells. Meanwhile, mTORC2 activates oxidative metabolism (157, 158).

In other way, long-lived specific memory cells that circulate between the secondary lymphoid organs, blood, and tissues do not proliferate significantly and have a quiescent functional state with scarce or no cytokine production. The main difference with naïve cells is that memory cells need to be prepared to respond quickly and efficiently to a new contact with the antigen. Their metabolism is based on the oxidation of glucose and fatty acids and they are characterized by a high content of large mitochondria, which are generated by fusion of the individual organelle that support the energetic requirements in reactivations (159, 160). In addition, ATP obtained from glucose oxidation is used to synthesize fatty acids, which in turn will be oxidized. The motive of this futile cycle could be the maintenance of the mitochondrial activity in order to be ready to respond quickly to specific-antigen re-stimulation (161–163).

Following multiple antigenic challenge, mainly in the case of chronic viral and tumor antigens and in situations of chronic inflammation, the specific T lymphocytes go through successive phases of clonal division, which as mentioned above, changes its degree of differentiation and, in parallel, its phenotype and functional capacity. In this stage, metabolic switching occurs in favor of an oxidative phenotype and is unambiguously associated with increased mitochondrial ROS production. On the other hand, inhibition of fatty acid oxidation decreases NADPH and glutation (GSH) levels. However, ROS levels increase, suggesting that control of fatty acid oxidation, in addition to PPP, regulates NADPH levels, which is essential to regenerate GSH pools from the glutathione disulfide (164, 165).

**Figure 4:**
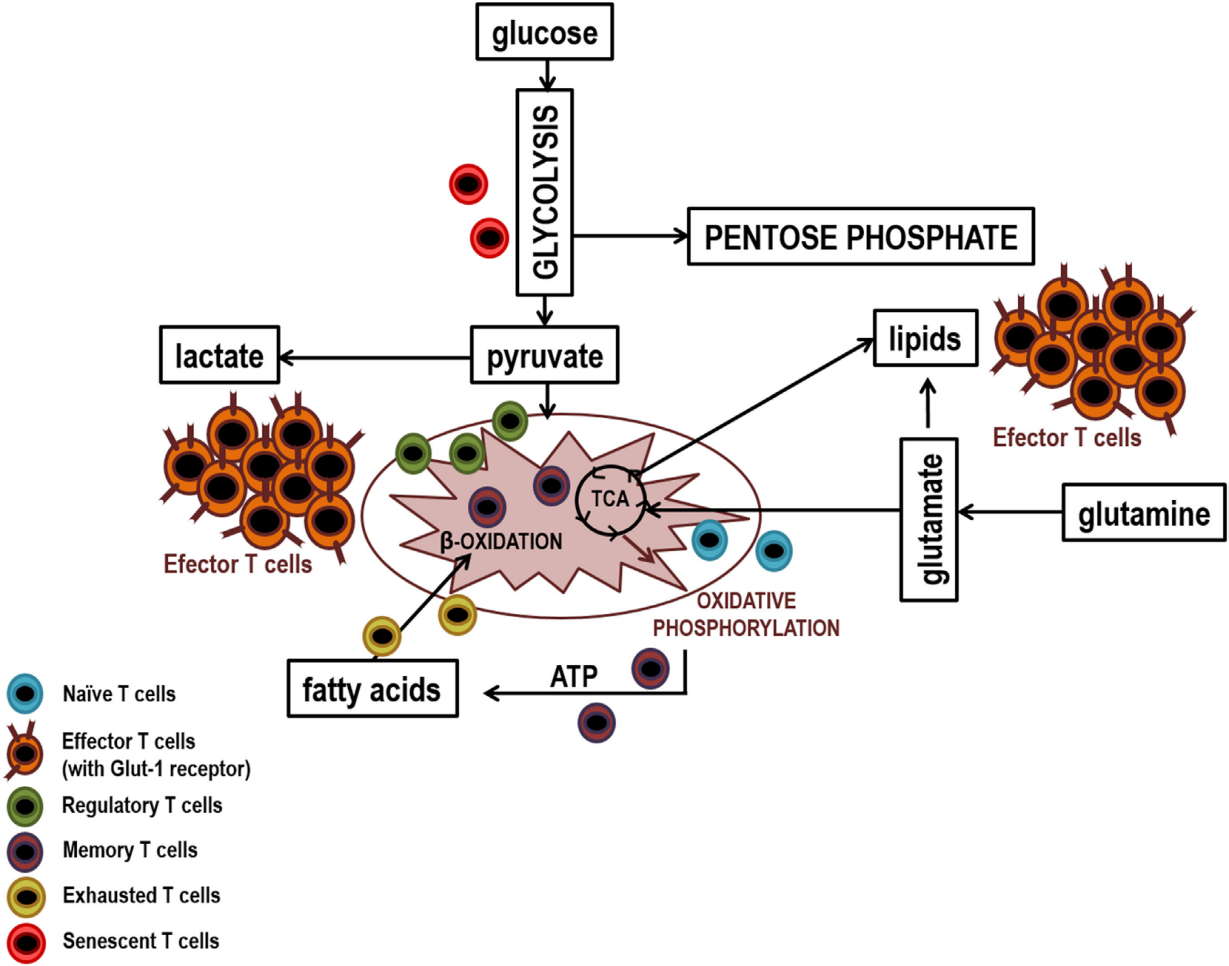
Main metabolic pathways of lymphocyte subpopulations. Naïve, memory, and regulatory T lymphocytes uptake low levels of glucose which it is metabolized by glycolysis and then the pyruvate generated is fully oxidized to CO_2_ in the mitochondria by the Krebs cycle. In contrast, effector lymphocytes have a large demand for glucose to maintain the biosynthetic processes that facilitate cellular growth, proliferation, and the synthesis of substantial quantities of effector molecules. Thus, effector lymphocytes show higher production of glucose transporters (GLUTs) and higher activity of glycolytic enzymes that facilitates elevated rates of glucose uptake and glycolytic flux. To sustain high rates of proliferation, pyruvate is converted to lactate and released out of cells. This prevents the accumulation of pyruvate which could result in the inhibition of the glycolysis pthway. Due to the high levels of glycolytic flux, glycolytic intermediates can be diverted into biosynthetic pathways to generate amino acids, lipids, and nucleotides in order to generate biomass. Senescent T lymphocytes use glycolysis extensively, partly because they have dysfunctional mitochondria and exhausted T lymphocytes mainly use lipid metabolism to carry out their poor cellular functions.

Finally, T lymphocytes reach replicative senescence, characterized by constitutive p38 mitogen-activated protein kinase activation, telomeric shortening, the loss of telomerase activity, and reduced proliferative capacity in response to stimulation (123). This new situation is also associated with metabolic adaptations. Again, a link between T lymphocytes aging and bioenergetic status has been proposed, since glucose deprivation in non-senescent T lymphocytes induces the activation of p38, and its constitutive activation inducer, the metabolic sensor of intracellular levels of ATP AMPK (5′-monophosphate activated protein kinase). These processes lead to a reduction in telomerase activity and proliferation similar to those observed in senescent T lymphocytes (166, 167). The main metabolic pathway used by these cells at this stage is glycolysis. Senescent T lymphocytes show mitochondrial dysfunction and consequently produce higher levels of ROS and defective mitochondrial biogenesis, which may justify their metabolic switch. P38 inhibition leads to mitophagy and thereby nonfunctional mitochondria are eliminated and ROS production is reduced. However, the increase in energy needed to sustain proliferation continues to be obtained from glycolysis and not from oxidative phosphorylation (168). As mentioned previously, high levels of inflammation in certain chronic viral infections and in cancer, induces an exhausted stage, similar to happen in senescence (113, 169, 170). There are numerous links between the inhibition of T lymphocytes by CTLA-4 and PD-1 and metabolic signaling pathways. CTLA-4 interacts with PP2A (protein phosphatase 2), a negative regulator of AKT, mTOR, and MAPK signaling, whereas PD-1 inhibits AKT phosphorylation by preventing CD28-mediated activation of PI3K (171). It has been shown that PD-1 inhibits glycolysis and amino acid metabolism and promotes lipid metabolism, whereas CTLA-4 inhibits both processes and mitochondrial biogenesis in memory cells (172, 173). Since the main metabolic pathway of T lymphocytes during activation is the aerobic glycolysis, these molecules could be blocking differentiation into effector T lymphocytes, at least partially, by metabolic regulation. The increase in β-oxidation of fatty acids could be an explanation for the maintenance of these cells, despite being exhausted, and in addition to its ability to recover functionality when interaction between PD-1 and its ligands hangs (173). Figure 4 shows a diagram of the main lymphocyte metabolic pathways in their different differentiation stages.

## Redox Control of Cellular Fate

Relationship between oxidative stress and inflammation has been widely documented (174). Oxidative stress plays a pathogenic role in many chronic inflammatory diseases. Lower levels of GSH, an intracellular thiol antioxidant, causes ROS production, which results in imbalanced immune response and inflammation. Moreover, protein oxidations turn into release of inflammatory signal molecules and inflammatory stimuli induce the release of peroxiredoxin 2, a redox-active intracellular enzyme (175).

Basal levels of ROS generated in response to endogenous and exogenous stimuli are crucial mediators of multiple cell processes such as growth, differentiation, or migration, but excessive production might induce cell death, apoptosis, and/or senescence. Oxidative stress triggered by the excessive ROS production, cause oxidative damage to cellular components such as DNA, proteins, or lipids, which is closely related to the pathogenesis of various diseases including cancer. In addition to exogenous ROS, major intracellular sources of ROS are NADPH oxidases and particularly mitochondria. Usually, physiologically generated ROS are balanced by non-enzymatic and enzymatic systems, such as, GSH, superoxide dismutases (SOD1/2), thioredoxins (Trx1/2), catalase, or peroxidases. In addition, NADPH, is one of the main thiol-dependent electron donors system in the cell and plays a critical role in the regulation of cellular redox environment and in a wide range of cellular pathways, including activation of transcription factors such as NF-κB, activator protein-1, p53, HIF-1, or the redox factor 1 (176). Elevated levels of ROS production have been considered an adverse event, playing an important role in tumor initiation and progression, but also in promoting inflammatory environments. However, ROS are now more widely recognized as important signaling molecules (177, 178). Redox signaling in cells by ROS such as hydrogen peroxide (H_2_O_2_) occurs through the reversible oxidation of cysteine thiol groups. A major cellular target of ROS is the thiol side chain (RSH) of cysteine, Cys sulfenic (Cys-SOH) and sulfinic (Cys-SO2H) acids have emerged as important mechanisms for regulation of protein function. These residues modifications result in reversible structural alterations that can modify protein function, which may imply either inactivation or gain of function (179).

The importance of ROS in immunity is exemplified by their generation and release in the form of an “oxidative burst” by phagocytic cells as part of the innate immune cell network to effectively destroy pathogens and clear debris. However, ROS exert, as it does in other cells, a dual role on T lymphocyte biology. Mild ROS are essential for T lymphocyte activation, expansion, and effector function (180–183). Still, elevated rate of ROS production or exposure and defective neutralization by antioxidant cellular systems, causes oxidative stress that compromises T lymphocyte proliferation and activity (182, 184). Balance between both situations may be fragile and the studies yield results that seem to be contradictory, probably due to different experimental conditions.

### Regulatory Effect of Oxidation on T Lymphocytes

Generation of ROS and Ca^2+^ release from intracellular stores are direct consequences of TCR/CD28 stimulation. Both are essential for TCR signaling, particularly in activation-induced CD95L expression (185). 5-lipoxygenase, NOX-2 and mitochondrial complexes are the most important sources of ROS in T lymphocytes (186–188). Oxidative signals originated from Complex I of the ETC regulate T lymphocyte activation-induced expression of IL-2 and IL-4, whereas Complex III is required for CD4+ activation and antigen-specific T lymphocyte expansion (189, 190). It is well known that ROS can activate the transcription factor NF-κB, whereas chronic exposure to oxidative stress inhibits its phosphorylation and the activation of T lymphocytes (191, 192). Related to this, translocation of NF-κB to the nucleus occurs in a cytoplasmic oxidative environment; however, binding to DNA requires reducing environment. Very high levels of ROS might affect both compartments and in such circumstances NF-κB pathway will be inhibited (193, 194). On the contrary, reduced ROS production is associated with decreased phosphorylation of JNK and NF-κB and, therefore, low IFN-γ and CD39 expression in CD8+ T lymphocytes (195). Equivalent results have been found in other regulatory pathways, since the exposure to low levels of ROS stimulates mTORC1 while high concentrations or long-term ROS treatment decrease mTORC1 activity (196).

Reactive oxygen species are also implicated in T lymphocyte differentiation, and murine models with specific knockouts of NOX-2, such as gp91^phox^ and p47^phox^, have been employed to test this association. p47^phox^ deficiency leads to Th17 differentiation, because mice p47^phox−/−^ have diminished expression of T-bet, STAT-1, and STAT-4 transcription factors, but increased phosphorylation of STAT-3. Additionally, it has been shown a reduced production of IL-2, IL-4, IFN-γ, TNF-α, and GM-CSF, but increased IL-10, IL-17, and TGF-β (188). On the contrary, lack of gp91^phox^ leads to a Th1 phenotype with reduced GATA-3 expression and STAT-5 and STAT-6 phosphorylation but increased T-bet expression. These T lymphocytes produce less IL-4 and IL-5 but more IL-17 and IFN-γ (189, 197). Then, NOX-2-deficienT lymphocytes showed a decreased in IL-4 but an increased IL-17 production. Interestingly, NOX-2 is not required for the proper activation of primary murine T lymphocytes, as gp91^phox−/−^ T lymphocytes have no defect in CD25 and CD69 expression, IL-2 production, or proliferation (187, 197).

CD4 T lymphocyte plasticity, switching from one lineage to another, may be affected by the oxidative microenvironment (37). As mentioned before, an oxidative microenvironment exerts opposite effects on cytokine secretion by Th1 compared to Th2 cells. When *in vitro* derived Th1 and Th2 clones or employed T lymphocytes derived from autoimmune thyroiditis to examine their ability to expand and produce cytokines in response to oxidative stress, low levels of H_2_O_2_ are able to reduce IFN-γ production by activated Th1 clones but to increase IL-4 secretion by activated Th2 clones (198). Besides, mitochondrial ROS can control T lymphocyte activation by upregulating IL-2 and IL-4 expression, and using T lymphocytes isolated from patients with atopic dermatitis, the inhibition of Complex I-mediated ROS blocks disease-associated spontaneous hyperexpression and TCR-induced expression of IL-4 (189).

### Oxidative Stress on T Lymphocytes

In opposite to regulatory role of mild oxidation, oxidative stress shows important effects during T lymphocyte development and differentiation. Thymus-specific elevation of mitochondrial superoxide (O2•−) disrupts normal T lymphocyte development and impairs the function of the mammalian adaptive immune system (199).

**Figure 5:**
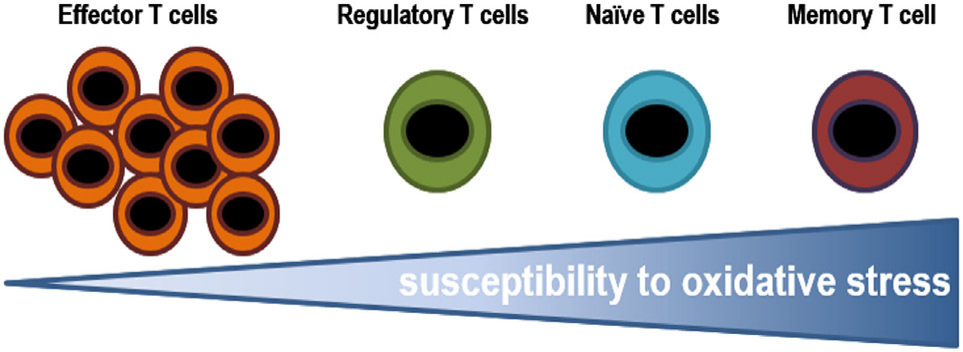
Susceptibility of T lymphocytes to oxidative stress. Effector cells are exposed to low oxidative environment, while memory cells are T lymphocytes found in the most oxidative environments. The ability of T lymphocytes to stand such oxidative conditions, in either an infectious disease state or a tumor microenvironment, might determine the pool of T lymphocytes.

The stage of differentiation largely determines sensitivity of individual T lymphocyte subsets to oxidative stress. The susceptibility of T lymphocytes to oxidative stress varies greatly depending on which stage of differentiation they are in (Figure 5). Effector cells are exposed to low oxidative environment, while memory cells are T lymphocytes found in the most oxidative environments. Some secreted cytokines can cause oxidative stress in T lymphocytes and in cancer cells. For example, tumors associated macrophages have been shown to induce sub-lethal oxidative stress in murine mammary cancer cells, maybe through the secretion of TNF-α. On the other hand, extracellular superoxide dismutases might finely tuning the levels of H_2_O_2_ in the extracellular milieu altering the proliferation and differentiation of immune cells (200). In fact, ROS may induce decreased viability in CD4+ T lymphocytes and the inhibition of DNA synthesis (181, 201). The latter is associated with alterations in the TCR signaling, including conformational changes of TCRζ and LCK, reduction of PLCγ-1 phosphorylation and calcium flux, and increased ERK phosphorylation. Moreover, it has been known that prolonged exposure to H_2_O_2_ suppress tyrosine phosphorylation, calcium flux, NFAT, and NF-κB activation, and IL-2 production (191).

*In vitro* assays testing the resistance of different subsets T lymphocytes to H_2_O_2_, it has been shown that it decreases from effector, to regulatory, naïve, and finally memory T lymphocytes (201). Effector T lymphocytes are able to withstand higher concentrations of ROS, which is probably essential to play their role in helping to phagocytes to eliminate pathogens (184). Whereas, human Tregs have higher thiol content and as a result, they are more resistant to cell death induced by H_2_O_2_ secreted by granulocytes than conventional T lymphocytes (201). Tregs suppress GSH synthesis and cysteine release by DCs in a CTLA-4-dependent manner. The resulting decrease in intracellular GSH leads to reduction in DNA synthesis in conventional T lymphocytes (191, 202), reduced levels leads to membrane displacement of LAT (central adapter protein in the TCR), and responsiveness T lymphocytes. A recent study showed that Tregs modulate GSH metabolism in T lymphocytes *via* cell contact and antigen-dependent, but not by an antigen-specific mechanism during suppression (202). The mechanism has been not identified yet but it could involve NADPH oxidase. Macrophages have also been shown to suppress T lymphocyte activation *in vitro* and *in vivo* through ROS (203) and, recent data demonstrates that macrophages induce Tregs *via* a ROS-dependent pathway that can be blocked by the NADPH oxidase inhibitor apocynin (204). In addition, scavenging enzymes that reduced oxidative intracellular milieu influence the regulation of T lymphocyte activity. Mitochondrial superoxide dismutase (MnSOD/SOD2) reduces T lymphocyte differentiation and functional ability by decreasing ROS levels (199, 205). Glutathione peroxidase-4 inhibits lipid peroxidation and plays a central role in the survival and the expansion of T lymphocytes.

In aging, the increase in oxidative stress and accumulated damage in the leukocytes appears to be related to the age-related deterioration of immune functions. A study by De la Fuente et al. reveals that the greater the cellular oxidative state and oxidative damage observed in immune cells of aged mice as well as in peripheral blood of elderly humans, were related with impaired immune responses (phagocytosis, chemotaxis, lymphoproliferation, etc.). However, in healthy centenarian individuals and in very long-lived mice, with preserved immune functions, they all presented a decreased expression of different inflammatory genes and highly controlled oxidative stress in their immune cells, which can partly explain their longevity (206–209). It is very important to emphasize in this context, that phagocytes, are postulated as the main responsible for oxidation-chronic inflammation stress that is associated with age and with immunosenescence (210). In the end, as a result of the oxidative damage that is related to aging, these cells could lose the ability to regulate their redox and inflammatory state, with the result of producing more and more oxidizing and inflammatory compounds, and thus contribute to the increase of oxidative and inflammatory stress. On the other hand, there are several studies performed on macrophages and peripheral blood neutrophils, in mice and humans, which have shown that these cells produce higher levels of oxidized compounds than those produced by lymphocytes and these levels increase with age. Furthermore, all these oxidative and inflammatory disbalances have been related to functional dysfunction of T lymphocytes.

On the other hand, different sources of ROS are involved in the activation-induced cell death (AICD) expression of Fas ligand (FasL) of T lymphocytes, and re-exposure to the specific antigen increases T lymphocyte sensitivity. First, H_2_O_2_ produced by DUOX-1 upon TCR serves to amplify proximal signaling events downstream of the TCR. Second, O 2•− released from mitochondrial Complex I, potentially in response to ERK signaling, triggers the expression of FasL. Finally, Fas ligation activates NOX-2, which probably contributes to the execution of the apoptotic program *via* H_2_O_2_-mediated activation of AKT and the inhibition of MEK. Moreover, cell-intrinsic antioxidants, such as glutathione, vitamin E, MnSOD, and CuZnSOD, interfere with FasL expression, thus counteracting AICD (184).

The selective cell death of effector cells with the memory phenotype may influence the size of the eventual memory T lymphocyte pool and consequently the functional ability of the responding cells. More specifically, CTLs exhibiting an EM phenotype were preferentially sensitive to AICD, compared CTLs with a CM. This increased sensitivity of EM T lymphocytes to TCR-induced AICD might correlate to the reduced levels of thiols in CD45RO+ T lymphocytes as compared to CD45RA+ memory T lymphocytes (211, 212). The loss of thiols after proliferation on repeated TCR stimulation may relate to apoptosis susceptibility. In this way, naïve T lymphocytes have higher levels of surface thiols and higher production of intracellular GSH compared to the antigen-experienced T lymphocytes (213–215). In addition, scavengers could reduce ROS-induced apoptosis of naïve and memory T lymphocytes. In fact, the increase levels of reduced thiol groups and intracellular GSH in a T lymphocyte subset could be responsible for its increased ability to persist in an oxidative stress microenvironment.

## Conclusion

Inflammation, both acute and chronic, both localized and systemic, plays a key role in the differentiation and ontogeny of T lymphocytes. From the moment of formation in the bone marrow until arrival to exhausted or senescent status, inflammation influences the development of T lymphocytes, and in turn the adaptive immune responses in the human body. The homeostasis and the way in which T lymphocytes respond to a specific antigen are influenced by the level of inflammation of the environment where the T lymphocytes are located. All the changes that occur along the ontogeny and differentiation of T lymphocytes require metabolic and oxidative adaptations. Despite the profound influence of inflammation in all these processes, little is known about the mechanisms through which it influences T lymphocytes; therefore, this is a research field with great practical applications to explore.

## Author Contributions

All authors have contributed equally to the elaboration of the manuscript.

## Conflict of Interest Statement

The authors declare that the research was conducted in the absence of any commercial or financial relationships that could be construed as a potential conflict of interest.
